# Investigating the function of F_c_‐specific binding of IgM to *P* *lasmodium falciparum* erythrocyte membrane protein 1 mediating erythrocyte rosetting

**DOI:** 10.1111/cmi.12403

**Published:** 2015-01-28

**Authors:** Liz Stevenson, Pie Huda, Anine Jeppesen, Erik Laursen, J. Alexandra Rowe, Alister Craig, Werner Streicher, Lea Barfod, Lars Hviid

**Affiliations:** ^1^ Centre for Medical Parasitology, Department of International Health, Immunology and Microbiology, Faculty of Health and Medical SciencesUniversity of CopenhagenCopenhagenDenmark; ^2^ Department of Infectious DiseasesCopenhagen University Hospital (Rigshospitalet)CopenhagenDenmark; ^3^ Niels Bohr Institute, Faculty of ScienceUniversity of CopenhagenCopenhagenDenmark; ^4^ Institute of Immunology and Infection ResearchUniversity of EdinburghEdinburghUK; ^5^ Liverpool School of Tropical MedicineLiverpoolUK; ^6^ Novo Nordisk Foundation Center for Protein Research, Faculty of Health and Medical SciencesUniversity of CopenhagenCopenhagenDenmark; ^7^ Novozymes A/SDK‐2880BagsværdDenmark

## Abstract

Acquired protection from *P* *lasmodium falciparum* malaria takes years to develop, probably reflecting the ability of the parasites to evade immunity. A recent example of this is the binding of the F_c_ region of IgM to VAR2CSA‐type PfEMP1. This interferes with specific IgG recognition and phagocytosis of opsonized infected erythrocytes (IEs) without compromising the placental IE adhesion mediated by this PfEMP1 type. IgM also binds via F_c_ to several other PfEMP1 proteins, where it has been proposed to facilitate rosetting (binding of uninfected erythrocytes to a central IE). To further dissect the functional role of F_c_‐mediated IgM binding to PfEMP1, we studied the PfEMP1 protein HB3VAR06, which mediates rosetting and binds IgM. Binding of IgM to this PfEMP1 involved the F_c_ domains Cμ3‐Cμ4 in IgM and the penultimate DBL domain (DBLζ2) at the C‐terminus of HB3VAR06. However, IgM binding did not inhibit specific IgG labelling of HB3VAR06 or shield IgG‐opsonized IEs from phagocytosis. Instead, IgM was required for rosetting, and each pentameric IgM molecule could bind two HB3VAR06 molecules. Together, our data indicate that the primary function of F_c_‐mediated IgM binding in rosetting is not to shield IE from specific IgG recognition and phagocytosis as in VAR2CSA‐type PfEMP1. Rather, the function appears to be strengthening of IE–erythrocyte interactions. In conclusion, our study provides new evidence on the molecular details and functional significance of rosetting, a long‐recognized marker of parasites that cause severe *P* *. falciparum* malaria.

## Introduction

Most *Plasmodium falciparum* infections in areas of stable parasite transmission produce only relatively mild symptoms or are asymptomatic. Nevertheless, about 600 000 people, mainly children, die from severe malaria complications annually (World Health Organization, 2013). It is not well understood why life‐threatening complications only develop in a minority of infections (Greenwood *et al*., 1991). A recent large‐scale study underscores the lack of clear relationships among parasite burden, number of previous episodes and disease severity (Goncalves *et al*., 2014). The marked concentration of severe and fatal malaria among young children is evidence that protective immunity can be acquired following natural exposure. However, even in areas of intense transmission of *P. falciparum* parasites clinical immunity takes years and often many disease episodes to develop, and protection is rarely if ever sterile. This piecemeal acquisition of protection appears to depend on gradual accumulation of IgG with specificity for a broad repertoire of variant antigens expressed on the infected erythrocyte (IE) surface (Marsh and Howard, 1986; Bull *et al*., 1998). Prominent among these is PfEMP1, a group of antigenically diverse high‐molecular weight proteins composed of modular DBL and CIDR domains, encoded by the *var* multi‐gene family that has about 60 members per parasite genome (Leech *et al*., 1984; Baruch *et al*., 1995; Smith *et al*., 1995; Su *et al*., 1995; Gardner *et al*., 2002). The PfEMP1 proteins are expressed on the IE surface knob protrusions and have affinity for host receptors in the vasculature (Baruch *et al*., 1995; Smith *et al*., 1995; Su *et al*., 1995). PfEMP1 expression allows IEs to sequester in various organs to avoid being cleared by the spleen (David *et al*., 1988). Development of severe disease is related to adhesion of IEs to particular receptors mediated by specific subsets of PfEMP1 proteins (Jensen *et al*., 2004; Salanti *et al*., 2004; Avril *et al*., 2012; Claessens *et al*., 2012; Lavstsen *et al*., 2012; Turner *et al*., 2013). An example is rosetting, which occurs when multiple uninfected erythrocytes bind to an IE forming aggregates that can impede the blood flow in vital organs. Rosetting has long been recognized but remains an incompletely understood IE adhesion phenotype that has been associated with malaria severity in some but not all studies (Carlson *et al*., 1990; Treutiger *et al*., 1992; Rowe *et al*., 1995; Kun *et al*., 1998). Many PfEMP1 proteins and a range of erythrocyte surface receptors have been implicated in this multifaceted phenotype, which can also involve additional, soluble components (reviewed by Mercereau‐Puijalon *et al*., 2008). Binding of IgM to IEs correlates with rosetting (Rowe *et al*., 2002), although IgM may be required, optional or irrelevant in the formation of various types of rosettes (Scholander *et al*., 1996; 1998; Clough *et al*., 1998; Vigan‐Womas *et al*., 2007; Le *et al*., 2008). In IgM‐dependent rosetting, only the pentameric form of IgM can facilitate the interaction between IEs and surrounding erythrocytes (Scholander *et al*., 1996). The interaction between IgM and PfEMP1 involves the F_c_ rather than the F_ab_ domains of IgM (Ghumra *et al*., 2008), and the role of IgM in rosetting therefore does not depend on its antigen specificity.

Although a correlation between rosetting and IgM binding is undisputed, exactly what role IgM plays in formation of rosettes is unclear. It has been proposed that IgM (and other serum factors) can act as ‘bridges’ between the IE and the surrounding erythrocytes, and IgM‐containing fibrillar strands have indeed been observed at apposed IE and erythrocyte membranes (Scholander *et al*., 1996). Alternatively, F_c_‐mediated IgM binding might serve an immune‐evasive function similar to that reported by us for the VAR2CSA‐type PfEMP1, which bind IgM but do not mediate rosetting (Creasey *et al*., 2003; Rasti *et al*., 2006; Barfod *et al*., 2011). The present study was designed to clarify the mechanism and functional significance of IgM in PfEMP1‐mediated rosetting.

## Results

### Recombinant HB3VAR06 constructs and *P* *. falciparum*‐IE selected for surface expression of HB3VAR06

**Figure 1:**
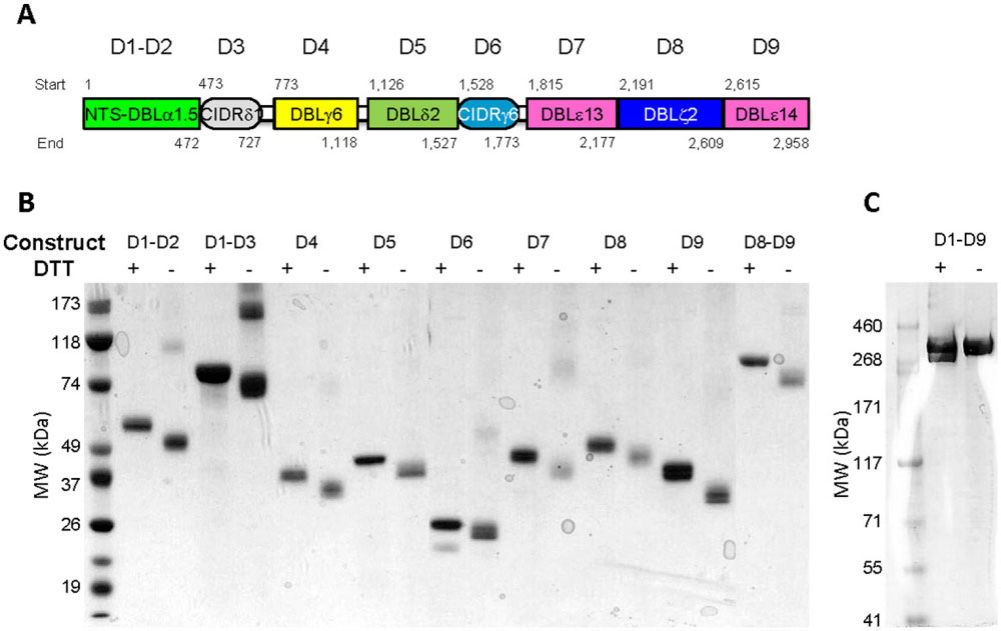
Recombinant HB3VAR06 constructs. Schematic representation of HB3VAR06 showing individual DBL and CIDR domains (domain start and end boundaries given above and below individual domains), named and colour coded as proposed by Rask *et al*. (2010) (A). Coomassie stain of an SDS‐PAGE with recombinant HB3VAR06 constructs under non‐reducing (+) and reducing (−) conditions (B and C).

**Figure 2:**
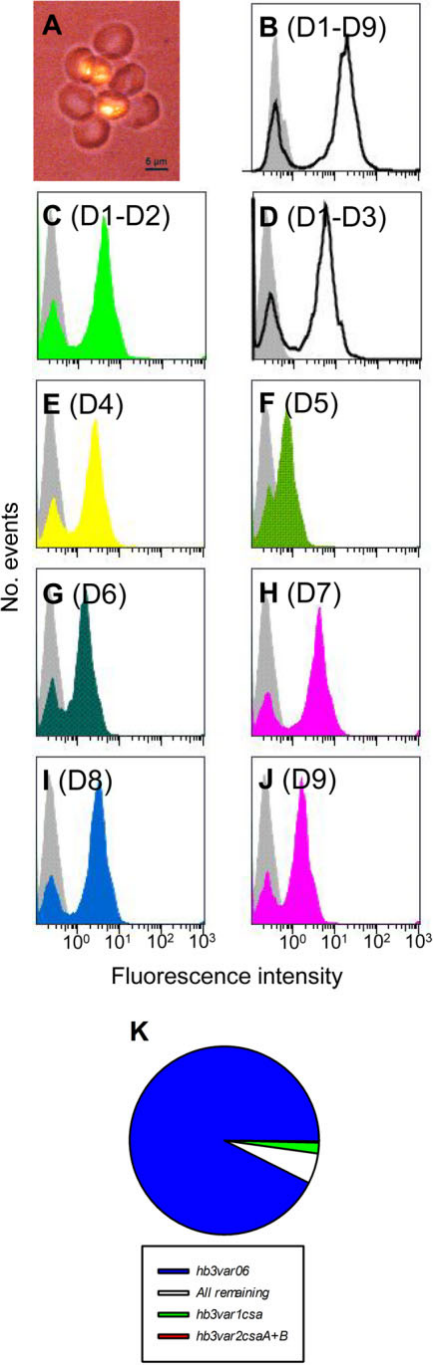
HB3VAR06‐positive *P* *. falciparum* parasites. Fluorescence micrograph of rosette around an erythrocyte infected by *in vitro*‐selected *P. falciparum* HB3. Error bar: 5 μm (A). Labelling of HB3VAR06^+^ IEs by antisera raised against different HB3VAR06 recombinant constructs measured by flow cytometry. Domains included in the constructs used for immunization are shown in brackets and background labelling (pre‐immunization sera) is shown by grey shading. Colour coding corresponds to that used in Fig. 1A, except for multidomain constructs including several domain subtypes (shown as black outlines) (B–J). Transcription profile of *var* genes in *P. falciparum* HB3 selected *in vitro* for expression of HB3VAR06 measured by quantitative real‐time PCR (K).

All recombinant HB3VAR06 constructs (overview in Fig. 1A) were secreted into supernatants predominantly as soluble monomers. In SDS‐PAGE, a shift in mobility was seen for all constructs following reduction, suggesting the presence of disulfide bonds in these cysteine‐rich proteins (Fig. 1B–C). *P. falciparum* HB3‐IEs selected *in vitro* for rosetting and IE surface expression of HB3VAR06 formed rosettes (Fig. 2A) and were labelled by all HB3VAR06‐specific antisera (Fig. 2B–J). Transcription analysis showed that *hb3var06* was the main *var* gene transcribed (93% of total *var* transcription) (Fig. 2K). No other single *var* gene accounted for more than 2% of total *var* gene transcription. Thus, our recombinant proteins, antisera and parasites had the expected characteristics; were specific; and were suitable for the present study.

### The binding of non‐specific IgM to HB3VAR06

**Figure 3:**
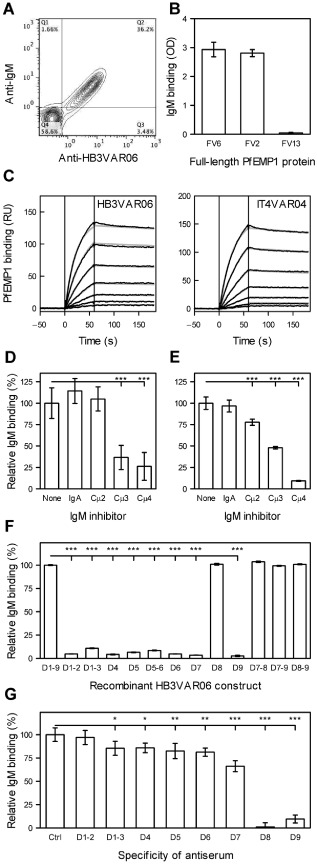
Binding of non‐specific IgM to HB3VAR06. Binding of non‐specific IgM and FV6‐specific antiserum to HB3‐infected IEs selected for expression of HB3VAR06 measured by flow cytometry of magnet‐purified late stage *P. falciparum* HB3‐infected erythrocytes (A). Binding of IgM to recombinant full‐length proteins representing HB3VAR06 (FV6), IT4VAR04 (FV2) and IT4VAR13 (FV13), respectively, measured by ELISA (B). Affinity of IgM for FV6 (left) and FV2 (right) measured by SPR. The SPR sensorgram data (black) and fits (grey) at five concentrations [1.125 (bottom trace); 2.25, 4.5, 9 and 18 nM (top trace)] are shown (C). Interference of non‐specific IgA and monoclonal antibodies specific for Cμ2 (HB57), Cμ3 (5D7) or Cμ4 (1G6) with IgM binding to HB3VAR06^+^ IEs measured by flow cytometry (D). Interference of IgA and monoclonal antibodies specific for Cμ2 (HB57), Cμ3 (5D7) or Cμ4 (1G6) with IgM binding to recombinant full‐length HB3VAR06 measured by ELISA (E). Binding of IgM to recombinant HB3VAR06 single‐, double‐ and triple‐domain constructs relative to IgM binding to FV6 measured by ELISA (F). Interference with IgM binding to HB3VAR06^+^ IEs by antisera raised against recombinant HB3VAR06 single‐ and double‐domain constructs measured by flow cytometry (G). Means and standard deviation and values statistically significant different (*P < 0.05; **P < 0.01; ***P < 0.001) from control values in the leftmost bar of each panel are shown (B, D–G). Control values were results obtained in the absence of any IgM inhibitor (D and E) using FV6 as the coating antigen (F) or in the presence of pre‐immunization serum (G). All experiments were repeated at least three times with similar results.

All HB3VAR06^+^ IEs bound non‐specific IgM (Fig. 3A) in agreement with an earlier report (Ghumra *et al*., 2012). Recombinant full‐length HB3VAR06 (FV6) and recombinant full‐length IT4VAR04 (a VAR2CSA‐type PfEMP1; FV2) both efficiently bound non‐specific IgM in enzyme‐linked immunosorbent assay (ELISA) in contrast to a third recombinant full‐length PfEMP1 (IT4VAR13; FV13), which mediates IE adhesion to ICAM‐1 and does not mediate rosette formation (Fig. 3B). The affinity of FV6 and FV2 for non‐specific IgM were both found by surface plasmon resonance (SPR) to be in the low nanomolar range (*K*_D_ = 0.1 and 0.3 nM, respectively), with rapid association (*k*_a_ = 2.3 × 10^6^ and 1.8 × 10^6^ M^–1^ s^–1^) and slow dissociation kinetics (*k*_d_ = 3.0 × 10^−4^ and 5.1 × 10^−4^ s^−1^) (Fig. 3C).

Several lines of evidence show that the interaction between non‐specific IgM and VAR2CSA‐type PfEMP1 involves the Cμ4 domain of IgM and the C‐terminal DBL domains in that type of PfEMP1 (Rasti *et al*., 2006; Semblat *et al*., 2006; Czajkowsky *et al*., 2010; Barfod *et al*., 2011). We found that antibodies specific for the IgM Cμ4 domain (and to a lesser extent Cμ3) inhibited binding of IgM to HB3VAR06^+^ IEs (Fig. 3D) and to FV6 (Fig. 3E). This finding corresponds with previous data regarding F_c_‐mediated IgM binding to other PfEMP1 proteins (Ghumra *et al*., 2008; Barfod *et al*., 2011). The site for F_c_‐mediated IgM binding in HB3VAR06 was identified in experiments with recombinant single‐, double‐ and triple‐domain constructs, which demonstrated that non‐specific IgM only bound to constructs containing DBLζ2 (domain D8) (Fig. 3F). In agreement with this, incubation of HB3VAR06^+^ IEs with antisera to the two C‐terminal HB3VAR06 domains D8 and D9 strongly inhibited the ability of non‐specific IgM to bind to the IEs, whereas the effect of the remaining HB3VAR06‐specific antisera was limited and decreased with increasing distance from D8 (Fig. 3F).

### F_c_‐mediated IgM binding to HB3VAR06 and immune evasion

**Figure 4:**
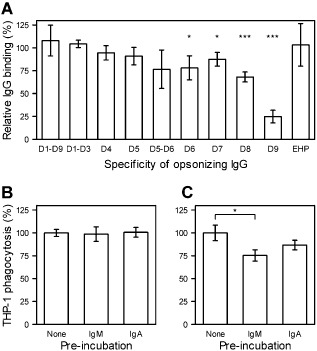
Non‐specific antibody interference with opsonization and phagocytosis. Interference with HB3VAR06 domain‐specific IgG recognition of HB3VAR06^+^ IEs by non‐specific IgM measured by flow cytometry (A). Phagocytosis of IEs opsonized by FV6‐specific antiserum (B) or human immune plasma (C) after or without pre‐incubation of IEs with non‐specific IgM or IgA measured by flow cytometry. Means and standard deviation, and statistically significant values (*P < 0.05; **P < 0.01; ***P < 0.001) relative to results obtained with pre‐immunization serum (A) or without IgM pre‐incubation (B and C) are shown.

**Figure 5:**
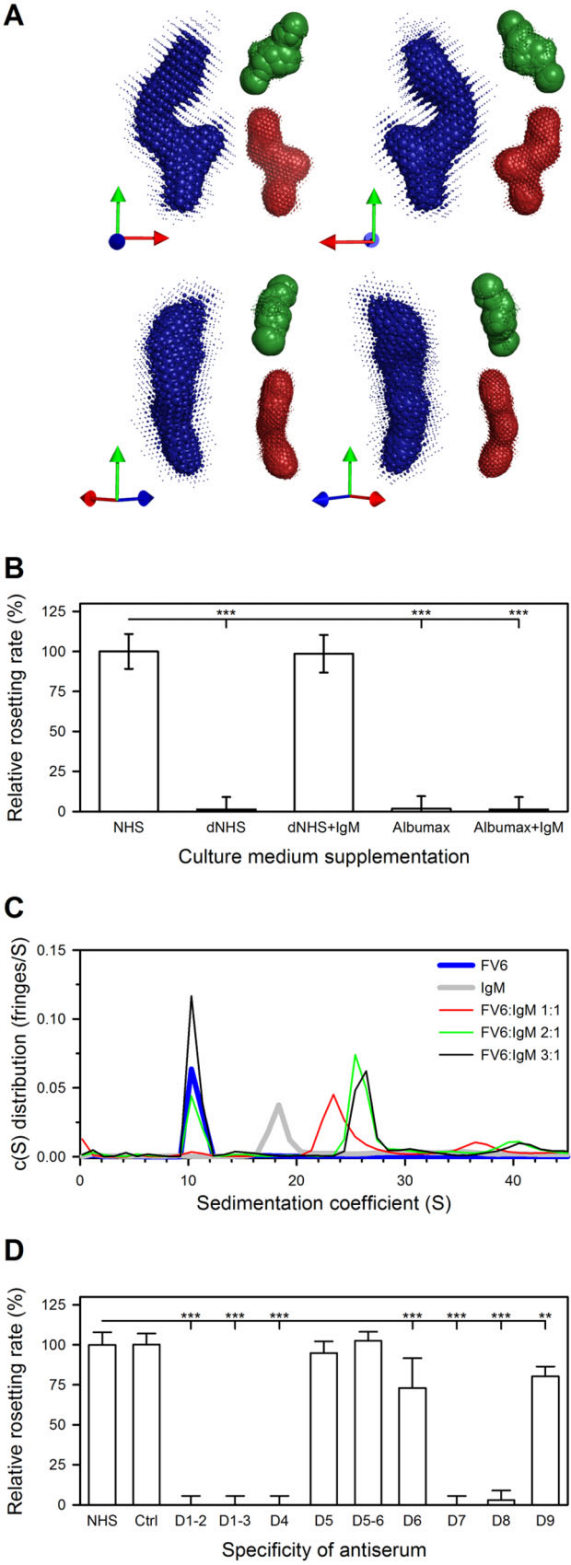
The three‐dimensional conformation of HB3VAR06, IgM dependency of rosetting and stoichiometry of the FV6:IgM interaction. SAXS modelling of the envelopes of FV6 (blue), the N‐terminal domains D1–D3 (green) and the C‐terminal domains D7–D9 (red) as seen from four different angles. The relative point of view in each panel is indicated by arrows in the bottom left panel corner (A). Rosetting rates of HBVAR06^+^ IEs in culture medium supplemented with non‐immune human serum (NHS), IgM‐depleted NHS (dNHS), dNHS plus IgM (dNHS + IgM), Albumax or Albumax plus IgM (Albumax + IgM) are presented as in Fig. 3 (B). Analytical ultracentrifugation of recombinant full‐length HB3VAR06 (FV6; blue) or IgM (IgM; grey), and of FV6 and IgM together at molar ratios of 1:1 (red), 2:1 (green) and 3:1(black) (C). Rosetting rates of HBVAR06^+^ IEs in the presence of HB3VAR06 domain‐specific antisera or pre‐immunization serum (Ctrl), relative to rosetting in NHS. Data presented as in Fig. 3G (D).

Based on the structural similarity of the interaction between non‐specific IgM on the one hand and HB3VAR06 and VAR2CSA‐type PfEMP1 on the other, we proceeded to examine the possibility of a corresponding functional similarity. We previously reported that F_c_‐mediated IgM binding to VAR2CSA‐type PfEMP1 has an immune‐evasive function as it interferes with antigen‐specific IgG recognition and inhibition of phagocytosis of IgG‐opsonized IEs (Barfod *et al*., 2011). Incubation of HB3VAR06^+^ IEs with IgM had no effect on the ability of IgG in FV6‐specific antiserum (Fig. 4A, leftmost bar) or in EHP (Fig. 4A, rightmost bar) to label the IEs. Antisera to the N‐terminal half (D1–D5) of HB3VAR06 were similarly unaffected by pre‐incubation with IgM, whereas IE reactivity of antisera to domains further downstream were increasingly inhibited by IgM incubation (Fig. 4A). These findings agreed well with the data pointing to DBLζ2 (D8) as the site for F_c_‐mediated binding of IgM to HB3VAR06. The reciprocal ability of antigen‐specific IgG and non‐specific IgM to interfere with each other (Fig. 3F–G) suggests that they have comparable binding affinities for HB3VAR06, similar to previous reports for VAR2CSA‐type PfEMP1 (Barfod *et al*., 2011). However, the data in Fig. 4A indicate that IgM would not efficiently inhibit phagocytosis of IgG‐opsonized HB3VAR06^+^ IEs because of its inability to shield N‐terminal IgG epitopes. This was confirmed experimentally as non‐specific IgM did not reduce phagocytosis of HB3VAR06^+^ IEs opsonized by FV6‐specific antiserum (Fig. 4B). Similarly, the effect of IgM on IEs opsonized by human immune plasma was small [25% (95% confidence interval: 8–42%)] and not different from that obtained with non‐specific IgA, which does not bind to HB3VAR06 (Fig. 4C). Finally, the IgM/IgG competition data (Fig. 3F–G) would be consistent with HB3VAR06 having an elongated, rod‐like structure causing the observed simple relationship between IgG epitope and IgM binding site proximity on the one hand, and shielding capacity of non‐specific IgM on the other. We confirmed this prediction by small‐angle X‐ray spectrometry (SAXS) analysis of FV6 (Fig. 5A, Supplementary Fig. S1, Table 1) yielding a low‐resolution structure and statistics similar to those obtained with the elongated FV13 (Brown *et al*., 2013) but different from those of the globular FV2 (Srivastava *et al*., 2010; Clausen *et al*., 2012). Additional SAXS analysis of N‐terminal (D1‐D3) and C‐terminal (D7‐D9) HB3VAR06 constructs (Fig. 5A) allowed orientation of the molecular SAXS envelope of FV6.

**Table 1:** Basic SAXS analysis statistics

	MW_Protein_ (*k*D)	MW_I(0)_ (*k*D)	*R*_g_ (nm)	*D*_max_ (nm)	I(0)_measured_	I(0)_theoretical_
FV6 (D1‐D9)	345	366	9.1	34.8	1.38	1.10
D1‐D4	85	84	4.0	14.1	0.124	0.11
D7‐D9	133	120	5.4	19.0	0.123	0.14

Theoretical molecular masses (MW_Protein_) are based on the amino acid sequences of recombinant protein constructs. SAXS‐derived molecular masses [MW_I(0)_] were calculated from the obtained scattering intensity [I(0)]. Particle radius of gyration (*R*_g_) calculations on Guinier analysis of the scattering data. Maximum particle size values (*D*_max_) were obtained from the derived pair–distance distribution functions. The theoretical I(0) was calculated from the amino acid sequence and protein concentration.

### F_c_‐mediated IgM binding to HB3VAR06 and rosetting

Rosetting is a highly variable phenotype, which depends on serum factors in some cases but not in others (Mercereau‐Puijalon *et al*., 2008). The factors involved in serum‐dependent rosetting have not been unequivocally identified, although IgM has been repeatedly implicated (Scholander *et al*., 1996; Clough *et al*., 1998; Somner *et al*., 2000; Rowe *et al*., 2002; Luginbuhl *et al*., 2007). We could confirm that serum and IgM is indeed required for rosetting of HB3VAR06^+^ IEs. Thus, rosetting did not occur in serum‐free Albumax‐containing culture medium and depletion of IgM abolished rosetting in serum‐containing medium (Fig. 5B). Rosetting was fully restored when exogenous IgM was added back to IgM‐depleted serum, whereas addition of exogenous IgM to Albumax medium had no effect (Fig. 5B). Thus, IgM appears to be necessary but is not in itself sufficient for rosette formation in *P. falciparum* HB3 expressing HB3VAR06.

As IgM binds near the C‐terminal, membrane‐proximal end of HB3VAR06 opposite the erythrocyte‐binding N‐terminal head structure, the effect of non‐specific IgM on rosetting could be indirect and related to its pentameric structure. Indeed, previous studies have shown that only intact IgM augments rosetting (Scholander *et al*., 1996; Somner *et al*., 2000; Ghumra *et al*., 2008). We therefore assessed the stoichiometry of the IgM–Fv6 interaction via analytical ultracentrifugation. Our experiments showed that FV6 existed predominantly as a monomer with a sedimentation coefficient of 10.5 S (Fig. 5C). Pentameric IgM was found to have a sedimentation coefficient of 18.5 S (Fig. 5C) in agreement with the literature (Miller and Metzger, 1966). Ultracentrifugation of FV6 and IgM mixed at equimolar (1:1) concentration yielded a single peak at 23.5 S, indicating that all FV6 and IgM were bound to each other and formed a single, larger complex. When FV6 was added in molar excess of IgM (2:1 and 3:1), two main peaks were evident, one at 26 S corresponding to two FV6 molecules bound to a single IgM molecule (2:1 complex) and one corresponding to free FV6. More unbound FV6 was seen in the 9S peak at the 3:1 than the 2:1 ratio, indicating that the IgM molecules could not accommodate more than two FV6 each (2:1 complex). Small additional peaks above 36 S were interpreted as aggregated species.

### Both ends of HB3VAR06 are required for rosetting

Ghumra *et al*. (2008) have documented the involvement of the N‐terminal head structure of HB3VAR06 in rosetting. To verify that finding, and to examine the involvement of the IgM‐binding DBL domain near the C‐terminus of the molecule, we assayed the effect of HB3VAR06 domain‐specific antisera on rosetting. We found that antisera specific for domains at the N‐terminus of HB3VAR06 (domains D1‐D4) as well as the C‐terminal domains D7 and D8 inhibited rosette formation, whereas antisera to the central domains D5 and D6 did not. Thus, epitopes at both the N‐ and the C‐terminus of this PfEMP1 are necessary for rosetting.

## Discussion

In this study, we provide a multi‐approach analysis of the molecular details and functional significance of F_c_‐dependent binding of IgM to a PfEMP1 involved in rosetting. The formation of rosettes of uninfected erythrocytes around *P. falciparum* IEs is a conspicuous and well‐recognized phenotype mediated by PfEMP1 (Chen *et al*., 1998) that has been repeatedly associated with severe malaria (reviewed by Mercereau‐Puijalon *et al*., 2008). We used HB3VAR06, previously identified as the dominant PfEMP1 protein expressed by *P. falciparum* HB3 selected for IgM‐dependent rosetting (Ghumra *et al*., 2012). We first generated a series of recombinant single‐domain, multiple‐domain and full‐length constructs representing HB3VAR06 (Fig. 1). Following repeated selection of IEs with corresponding antisera, the HB3VAR06‐encoding gene *hb3var06* was the predominant *var* gene transcribed by the parasites, and the large majority of IEs formed rosettes and was labelled by each of the HB3VAR06‐specific antisera and bound non‐specific IgM (Figs 2 and 3A). Recombinant full‐length HB3VAR06 (FV6) bound IgM to the same extent (Fig. 3B) as the VAR2CSA‐type PfEMP1 that are involved in placental sequestration of IEs but do not mediate the formation of rosettes (Creasey *et al*., 2003; Salanti *et al*., 2003; 2004; Rasti *et al*., 2006). The affinity of the interaction between FV6 and IgM (Fig. 3C) was in the same range as those reported for the interaction between other full‐length PfEMP1 proteins and their cognate receptors (Khunrae *et al*., 2010; Srivastava *et al*., 2010; Brown *et al*., 2013). This high‐affinity interaction between individual FV6 molecules in solution and immobilized IgM effectively rules out the previously suggested theory that only pentameric IgM supports rosetting because it is required to overcome an inherently low affinity between IgM and DBL domains in PfEMP1 (Scholander *et al*., 1996; Ghumra *et al*., 2008). The interaction between IgM and HB3VAR06 involved Cμ4 in IgM and the penultimate DBLζ2 domain at the C‐terminus of HB3VAR06 (Fig. 3D–G). This is very similar to what has been reported previously for VAR2CSA‐type PfEMP1 and for another rosette‐mediating PfEMP1 protein, TM284VAR1 (Rasti *et al*., 2006; Semblat *et al*., 2006; Ghumra *et al*., 2008; Czajkowsky *et al*., 2010; Barfod *et al*., 2011).

At the outset of the present study, the functional significance of the interaction of HB3VAR06 with IgM remained unclear. We therefore initially hypothesized that binding of non‐specific IgM to rosetting PfEMP1 might shield IEs from specific IgG recognition and phagocytosis as reported for IEs expressing VAR2CSA‐type PfEMP1 (Barfod *et al*., 2011), as well as in other host–parasite systems (Garcia *et al*., 1997; Echaide *et al*., 1998). Alternatively, or in addition, the IgM might act as ‘bridges’ between the central IE and the surrounding erythrocytes in rosettes (Scholander *et al*., 1996). However, we found that non‐specific IgM binding to HB3VAR06 was inefficient in inhibiting IgG recognition of HB3VAR06 and phagocytosis of IgG‐opsonized IEs, except in the case of IgG specific for epitopes near the IgM binding site in the DBLζ2 domain of this PfEMP1 (Fig. 4). The inability of non‐specific IgM to shield HB3VAR06 from specific IgG recognition agrees with our demonstration that this PfEMP1 has an elongated, rod‐like structure (Fig. 5A) similar to that of the ICAM‐1‐binding PfEMP1 protein IT4VAR13 (Brown *et al*., 2013). A Kratky plot of the FV6 SAXS data (Supplementary Fig. S1C) furthermore indicated that the HB3VAR06 ectodomain is rigid as reported for other PfEMP1 molecules (Brown *et al*., 2013; Higgins and Carrington, 2014). This is the first structural description of a full‐length PfEMP1 protein mediating rosetting, and it provides a plausible explanation for the divergent functional consequences of IgM binding to HB3VAR06 and the much more globular VAR2CSA‐type PfEMP1.

HB3VAR06‐dependent rosetting was found to require non‐specific IgM (Fig. 5B) binding to HB3VAR06 at the C‐terminal end of the PfEMP1 (Fig. 3F–G). In contrast, the interaction with the surrounding erythrocytes involves the N‐terminal head structure (Rowe *et al*., 1997; Chen *et al*., 2000; Ghumra *et al*., 2012) at the opposite end of this extended molecule that is more than 30 nm long (Table 1). Together, these observations make it unlikely that IgM can act as ‘bridges’ between the IE and the surrounding erythrocytes in a rosette (Scholander *et al*., 1996), unless additional and so far unidentified components are involved. This latter possibility would be consistent with earlier evidence (Scholander *et al*., 1998; Somner *et al*., 2000) and our finding that addition of purified non‐specific IgM to serum‐free medium did not lead to rosette formation (Fig. 5B). In any case, our evidence demonstrates the rosetting mediated by HB3VAR06 involves structural elements at both the N‐ and the C‐terminal ends of this PfEMP1 (Fig. 5D).

Based on the above findings, and the fact that only pentameric IgM can support rosetting (Scholander *et al*., 1996; Somner *et al*., 2000; Ghumra *et al*., 2008), we therefore speculated that the function of IgM in rosetting might instead be to facilitate coordinated interaction of multiple PfEMP1 head structures with their receptors on adjacent erythrocytes (Rowe *et al*., 1995; Barragan *et al*., 2000a, 2000b; Vogt *et al*., 2004). Although rosetting is a highly variable phenotype, rosettes can consistently be disrupted by sulfated glycosaminoglycans (Carlson and Wahlgren, 1992; Rowe *et al*., 1994; Barragan *et al*., 1999), whereas they can form even after pretreatment of the uninfected erythrocytes by protease (Rowe *et al*., 1994). This suggests that rosetting PfEMP1 proteins interact mainly with carbohydrate moieties on the surrounding erythrocytes. The affinity of the rosetting PfEMP1 VarO head structure for blood group trisaccharides has previously been found to be in the micromolar range (Vigan‐Womas *et al*., 2012), and coordinated interaction involving multiple well‐aligned and knob‐associated PfEMP1 molecules might therefore be required to increase avidity sufficiently for rosetting to occur. It is not known how many PfEMP1 molecules are expressed per knob, but theoretical estimates are in the 10–100 range (Joergensen *et al*., 2010b), and alignment of these PfEMP1 molecules would potentially greatly increase their combined avidity for host receptors. Such a function would resemble that proposed for the malaria parasite proteins *P. falciparum* erythrocyte‐binding antigen‐175 and *P. vivax* Duffy binding protein (Tolia *et al*., 2005; Batchelor *et al*., 2011; 2014; Wanaguru *et al*., 2013). In those, DBL domains form homo‐dimers when binding to their host receptors presumably to enhance receptor affinity and specificity, thereby facilitating erythrocyte invasion. Although the parasite molecule and mechanism we propose here differ from that already demonstrated for merozoite‐expressed adhesins, the concept of multimerization to increase avidity for erythrocyte receptors is a mechanism already employed by malaria parasites.

In conclusion, our study provides comprehensive new evidence on the molecular details and functional significance of F_c_‐dependent IgM binding in rosetting, which is a long‐recognized marker of parasites causing severe *P. falciparum* malaria. The interaction between non‐specific IgM and the rosette‐mediating PfEMP1 protein HB3VAR06 involves the same F_c_ domains in IgM as those interacting with VAR2CSA‐type PfEMP1. Nevertheless, the function appears to be markedly different as F_c_‐dependent IgM binding in rosetting does not protect the parasite from phagocytosis and is required for parasite adhesion. Furthermore, our data suggest that the influence of IgM on rosetting is related to its capacity to bind multiple PfEMP1 proteins, potentially increasing the combined avidity of multiple PfEMP1 proteins for erythrocyte carbohydrate receptors. We cannot formally rule out that IgM can form ‘bridges’ between IEs and surrounding erythrocytes, but our data suggest that structural limitations would make this unlikely at least for IgM alone. The identity and role of additional serum proteins in HB3VAR06‐mediated rosetting, such as fibrinogen, albumin, von Willebrand factor and TSP, which have all previously been implicated in rosetting (Treutiger *et al*., 1999) remain an open question requiring further study.

## Experimental procedures

### Ethics statement

The collection of human plasma samples was approved by the Institutional Review Board of Noguchi Memorial Institute for Medical Research, University of Ghana (Study Number 038/10‐11), and by the Regional Research Ethics Committees, Capital Region of Denmark (Protocol H‐4‐2013‐083). All donors were adults and provided written informed consent. All the animal experiments were conducted according to Danish Law and approved (permit 2012‐15‐2934‐00567) by the Danish Animal Procedures Committee (‘Dyreforsøgstilsynet’).

### Recombinant parasite proteins

The entire ectodomain of the PfEMP1 protein HB3VAR06 (FV6) was codon optimized (GenBank accession number KP203835) for insect cell expression by GeneArt (Regensburg, Germany). Single‐, double‐, triple‐domain and full‐length constructs were cloned into the transfer vector pAcGP67‐A (BD Biosciences, San Jose, CA, USA) with a C‐terminal hexa‐histidine tag. The transfer vector was then co‐transfected with linearized BakPak6 Baculovirus DNA (BD Biosciences) into Sf9 insect cells to generate recombinant virus particles. Secreted proteins were purified from the supernatants of transfected high‐five insect cells using Ni^2+^ metal chelate agarose HisTrap HP columns (GE Healthcare, Fairfield, CT, USA) followed by dialysis of eluates (20 mM Na_2_PO_4_, 500 mM NaCl, pH 7.2). Proteins used for analysis by SPR, analytical ultra‐centrifugation or small‐angle X‐ray spectrometry (SAXS) were further purified on Superdex 200 16/60 size exclusion chromatography columns (GE Healthcare). Domain boundaries for all the recombinant HB3VAR06 constructs are shown in Fig. 1. Two other recombinant full‐length PfEMP1 proteins described previously, IT4VAR13 (FV13; ref. Brown *et al*., 2013) and the VAR2CSA‐type PfEMP1 protein IT4VAR04 (FV2; ref. Khunrae *et al*., 2010), were included as control antigens.

### Animal antisera and human plasma

Antisera specific for recombinant HB3VAR06 constructs were generated by subcutaneous immunization of rats (20 μg antigen in Freund's complete adjuvant followed by 20 μg in Freund's incomplete adjuvant 21 and 42 days later) and rabbits (50 μg antigen in Freund's complete adjuvant followed by 50 μg in Freund's incomplete adjuvant 28, 49 and 63 days later). One (FV6) or two (all other constructs) animals were used per construct for immunization. Antisera were collected on day 49 (rats) or 70 (rabbits), pooled and depleted of non‐specific O Rh^+^ erythrocyte reactivity. Pre‐immunization serum was used as negative control. Mouse monoclonal antibodies specific for human F_c_μ2, F_c_μ3 and F_c_μ4 (described by Rudich *et al*., 1985) were a kind gift from Patricia Mongini.

Pooled plasma from 10 anonymous healthy, *P. falciparum*‐exposed adults (exposed human plasma; EHP) or pooled serum from non‐exposed blood bank donors; NHS) was used with or without IgM depletion by incubation with biotinylated donkey anti‐human IgM‐F_c_μ5 (Jackson ImmunoResearch) coupled to streptavidin‐conjugated Dynabeads (MyOne T1, 8 mg; Life Technologies). Absence of IgM after depletion was confirmed by immunoblotting with horse radish peroxidase‐conjugated rabbit anti‐human IgM (1:1000; Dako).

### Measurements of IgM‐ and antigen‐specific IgG binding to PfEMP1

Binding of IgM to recombinant HB3VAR06 constructs was quantified by ELISA. Flat‐bottomed 96‐well MaxiSorp plates (Thermo Scientific) were coated overnight with recombinant protein (18 nM, 4°C) in Tris saline magnesium (TSM) buffer (20 mM Tris, 150 mM NaCl, 2 mM CaCl_2_, 2 mM MgCl_2_, pH 7.4), blocked [2 h, room temperature (RT)] with TSMB (TSM buffer with 1% Ig‐free BSA and 0.05% Tween‐20), washed with TSM buffer supplemented with 0.05% Tween‐20 and incubated (2 h, RT) with purified human IgM (Sigma; I8260; 10 nM in TSMB buffer). After washing away unbound antibody, IgM was detected using rabbit anti‐human IgM HRP (Dako; P215, 1:1000 in TSMB buffer). After washing unbound secondary antibody, bound HRP was reacted with OPD (Dako; S2045) according to manufacturer's instructions and absorbance detected (492 nm). In assays to determine which F_c_ domains were involved in IgM binding to HB3VAR06, IgM (10 nM) was pre‐incubated overnight (4°C) with mouse anti‐human F_c_μ domain‐specific monoclonal antibodies or IgA (IgA (Sigma; L1010; 100 nM) in TSMB buffer.

The binding of IgM and antigen‐specific IgG to IEs was detected by flow cytometry essentially as described (Barfod *et al*., 2011). In brief, late‐stage IEs were purified by magnet‐activated cell sorting (MACS) and labelled (1 × 10^5^ IEs, 30 min, RT) first with non‐specific IgM (10 nM), then rat or rabbit antisera or EHP (1:20), and finally appropriate secondary antibody (donkey anti‐human IgM‐PE (Jackson ImmunoResearch; 709‐116‐073; 1:400), goat anti‐rat IgG‐FITC (Life Technologies; clone 62‐9511; 1:150), goat anti‐rabbit IgG‐FITC (Vector; clone FI‐1000; 1:150) and ethidium bromide (2 μg ml^−1^). In some assays, the antibody order was reversed, i.e. first antisera or EHP, then IgM. In antibody competition assays, the competing antibodies were applied in separate steps. In experiments assessing IE surface labelling by non‐specific IgM and antigen‐specific IgG at the same time, the donkey anti‐human IgM‐PE and donkey anti‐rabbit PerCP (Jackson; 711‐126‐152; 1:50) were used as secondary antibody reagents and ethidium bromide was omitted. IEs were washed three times between incubation with primary and secondary antibody. Antibody surface labelling of IEs was quantified by flow cytometry using a Beckman Coulter FC500 instrument (Beckman Coulter) followed by analysis of list mode data files using FlowJo software v.7.6 (Treestar).

### Malaria parasite cultivation and *in vitro* selection procedures

*Plasmodium falciparum* HB3 parasites (Bhasin and Trager, 1984) were grown *in vitro* in O Rh^+^ erythrocytes using AlbuMax II (Life Technologies)‐supplemented Roswell Park Memorial Institute (RPMI) medium and a controlled atmosphere, essentially as described (Cranmer *et al*., 1997). IEs were selected for rosetting using sedimentation in gelatin two times per week as described (Handunnetti *et al*., 1992) and for surface expression of HB3VAR06 by immuno‐magnetic selection once every 1–2 weeks using HB3VAR06‐specific antisera and protein A‐coupled DynaBeads as described (Staalsoe *et al*., 2003). Cultures were kept synchronous by sorbitol treatment twice weekly as described (Moll *et al*., 2008). The genotypic identity of the parasites and absence of *Mycoplasma* contamination was verified regularly as described (Bengtsson *et al*., 2013).

### Var gene transcription analysis

*Plasmodium falciparum* RNA was prepared from ring stage IEs, reverse‐transcribed and used to determine the relative proportions of individual *var* gene transcripts by quantitative real‐time PCR as described in detail elsewhere (Joergensen *et al*., 2010a) using real‐time PCR‐optimized, gene‐specific primers (20 μM) for each of the *var* genes in the *P. falciparum* HB3 genome (Soerli *et al*., 2009).

### Rosetting assay

Rosetting assays were performed using cultures with high rosetting rates (60–95%) and frequencies were assessed by counting 200 ethidium bromide‐stained IEs and noting those that had two or more erythrocytes adhering using wet slide preparations and fluorescence microscopy. To determine the role of F_c_‐mediated IgM binding in rosetting, synchronous ring stage IEs were incubated overnight (37°C, 5% CO_2_) with NHS (10%), IgM‐depleted NHS, IgM‐depleted NHS plus human IgM (Sigma; 4 mg ml^−1^), Albumax II (10%) or Albumax II (10%) plus IgM (4 mg ml^−1^). The following day the relative rosetting frequency of triplicate wells was assessed as described above. To examine the involvement of different HB3VAR06 domains in rosetting, synchronous HB3VAR06^+^ IEs were grown from the ring to the late trophozoite stages in 10% NHS in the presence of 1:20 dilutions of domain‐specific antisera followed by assessment of rosetting rates as above.

### SPR assay

We used a Biacore 2000 (GE Healthcare) for affinity measurements by SPR. All experiments were performed in N‐[2‐hydroxyethyl]piperazine‐N′‐[2‐ethanesulfonic acid] (HEPES) buffer [0.01 M HEPES, 0.15 M NaCl, 3 mM ethylenediaminetetraacetic acid (EDTA), 0.005% v/v Surfactant P20] at pH 7.4 and 20°C. FV6 or FV2 were flowed over (50 ml min^−1^ for 60 s followed by buffer for 120 s) IgM (750 RU) immobilized on a CM4 biosensor chip (GE Healthcare) by amine coupling. After each run, the chip was regenerated (1 M NaCl). Specific binding response to IgM was calculated by subtracting the response to an uncoupled chip and to a buffer injection. The kinetic sensorgrams were fitted to a global 1:1 interaction model to allow calculation of *k*_a_, *k*_d_ and *K*_D_ using BIAevaluation software v.4.1 (GE Healthcare).

### Phagocytosis assay

Phagocytosis of antibody‐opsonized IEs was measured as described previously (Tippett *et al*., 2007). In brief, MACS‐purified late‐stage IEs were opsonized with FV6 antiserum or human immune‐serum (1:20 dilution, 30 min, RT), washed in PBS with 2% FBS (PBS2), labelled with ethidium bromide (0.1 mg ml^−1^, 10 min, RT), washed four times in PBS2 and resuspended in RPMI supplemented with 2% FCS. Human monocytic leukaemia line THP‐1 cells (TIB‐202; LGC Standards) (4 × 10^5^ per well) and IEs (2 × 10^5^ per well) were incubated (30 min, 37°C, 5% CO_2_) in 96‐well plates (Corning; 113 135 95). Non‐phagocytosed IEs were lysed (15 mM NH_4_Cl, 10 mM NaHCO_3_, 1 mM EDTA, 3 min), and phagocytosis assessed by flow cytometry as above. In some experiments, IEs were pre‐incubated with 10 nM IgM or IgA before addition of anti‐ or immune‐serum.

### SAXS

SAXS data on recombinant HB3VAR06 constructs were collected at the P12 BioSAXS beam line (PETRAIII). The beam line was equipped with a PILATUS 2M detector set 3.1 m from the sample using a 0.13–0.14 nm beam wavelength. Concentration series of different constructs were prepared in order to eliminate structure factor effects. All measurements were performed at 10°C, collecting 20 frames of 0.05 s exposure time of each construct. The pair distance distribution function [P(r)] was obtained by indirect Fourier transformation of the scattering data giving the estimated radius of gyration (*R*_g_) and maximum particle dimension (*D*_max_) (Svergun, 1992). For each construct, 20 *ab initio* bead models were generated from the scattering data using online DAMMIF (Franke and Svergun, 2009). The final models were created by averaging and filtering the 20 constructs using DAMAVER and DAMFILT (Volkov and Svergun, 2003).

### Analytical ultracentrifugation

Sedimentation velocity experiments were conducted using a Beckman Optima XL‐I analytical ultracentrifuge (Beckman Coulter) (40 000 r.p.m., 20°C). All the size exclusion chromatography‐purified protein samples were prepared in a PBS buffer (137 mM NaCl, 2.7 mM KCl, 10 mM Na_2_HPO_4_, 1.8 mM KH_2_PO_4_). Reference and sample were loaded into a double‐sector centrepiece and mounted in a Beckman An‐60 Ti rotor. Results from multiple scans (monitored at 280 nm) at molar ratios 1:1, 1:2 and 1:3 of IgM and FV6 (where the IgM concentration was kept constant at 0.2 μM to give an absorbance of 0.296 in a 1.2 cm path length) were fitted to a continuous size distribution using SEDFIT v.14.1 software (Schuck, 2000). Solvent density (1.00564 g ml^−1^), viscosity (0.01020 P) and the partial‐specific volumes of IgM (0.727 cm^3^ g^−1^) and FV6 (0.727 cm^3^ g^−1^) were calculated using SEDNTERP software (v.20120828) (Laue *et al*., 1992).

### Statistical analyses

All experiments were performed at least three times with similar results. Summary data in Figs 3, 4, 5 are reported as the overall means and standard deviations of all experiments performed expressed as a percentage of the relevant experimental control data. The statistical significance levels reported in Fig. 3D–G, Fig. 4B–C, Fig. 5B and Fig. 5D were obtained by one‐way analysis of variance followed by Holm–Sidak post hoc test to identify groups significantly different from the indicated control group. The data presented in Fig. 4A were analysed by Student's *t*‐test.

## Supporting information


**Fig. S1.** SAXS analysis of HB3VAR06. Theoretical scattering curves calculated from *ab initio* reconstructions (lines) and experimental scattering data (points and error bars) (A) and distance distribution functions (B) for full‐length HB3VAR06 (FV6, blue), the N‐terminal domains D1–D4 (red) and the C‐terminal domains D7–D9 (green) (B). The P(r) functions were calculated from the scattering intensity I(q). Kratky plot of full‐length HB3VAR06 (FV6) (C).
