# Supplementary figures and images for: Investigating the function of Fc‐specific binding of IgM to P lasmodium falciparum erythrocyte membrane protein 1 mediating erythrocyte rosetting
Source: Cell Microbiol. 2015 Jan 28;17(6):819–31. doi: 10.1111/cmi.12403 (PMC4737123; doi:10.1111/cmi.12403)

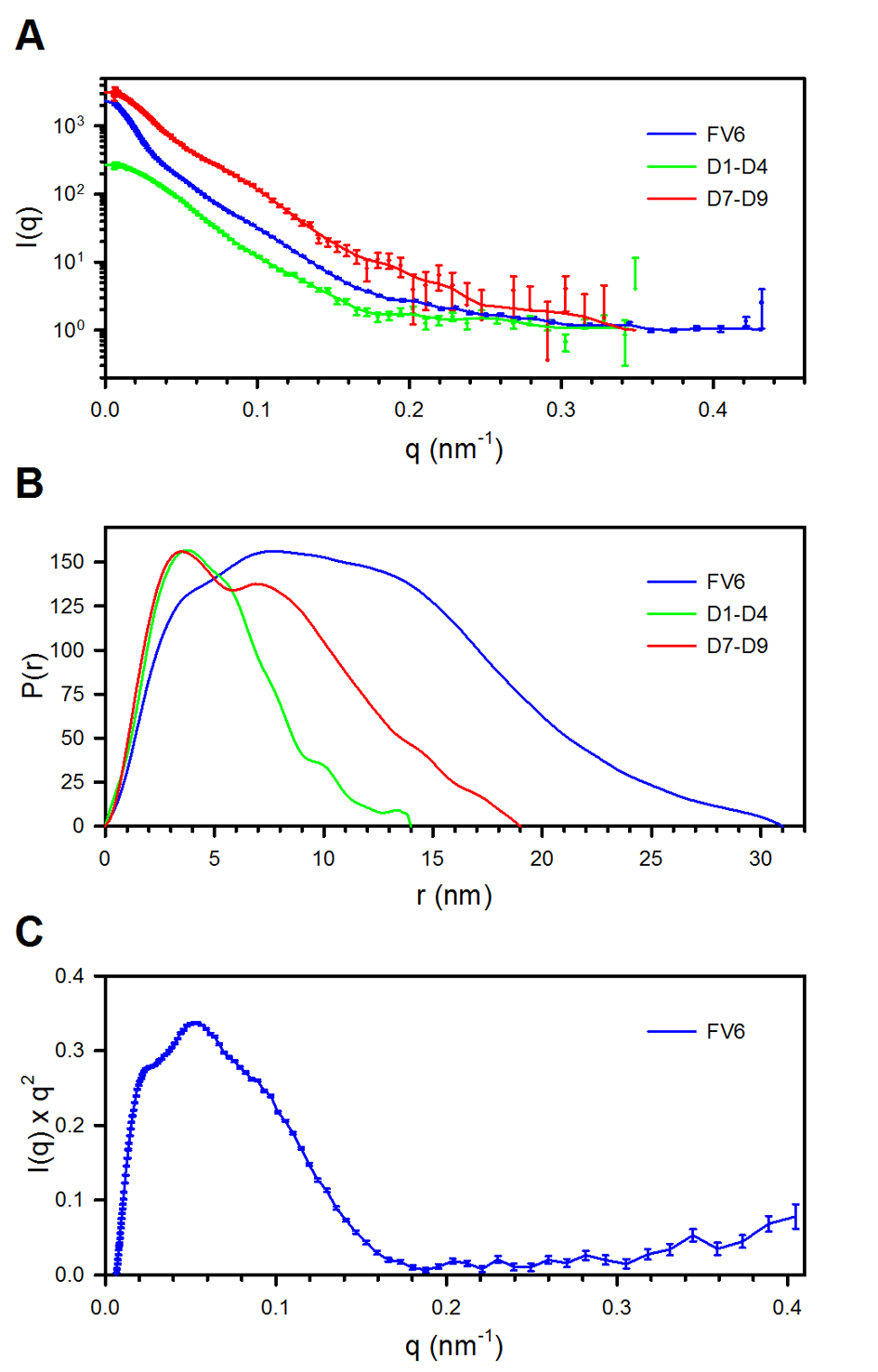

Supplement: Supplementary file 1 — Fig. S1. SAXS analysis of HB3VAR06. Theoretical scattering curves calculated from ab initio reconstructions (lines) and experimental scattering data (points and error bars) (A) and distance distribution functions (B) for full‐length HB3VAR06 (FV6, blue), the N‐terminal domains D1–D4 (red) and the C‐terminal domains D7–D9 (green) (B). The P(r) functions were calculated from the scattering intensity I(q). Kratky plot of full‐length HB3VAR06 (FV6) (C). [file CMI-17-819-s001.tif]
